# Supplementary material for: Light prevents pathogen-induced aqueous microenvironments via potentiation of salicylic acid signaling
Source: Nat Commun. 2023 Feb 9;14:713. doi: 10.1038/s41467-023-36382-7 (PMC9911384; doi:10.1038/s41467-023-36382-7)
Supplement: Supplementary file 1 — Supplementary Information [file 41467_2023_36382_MOESM1_ESM.pdf]

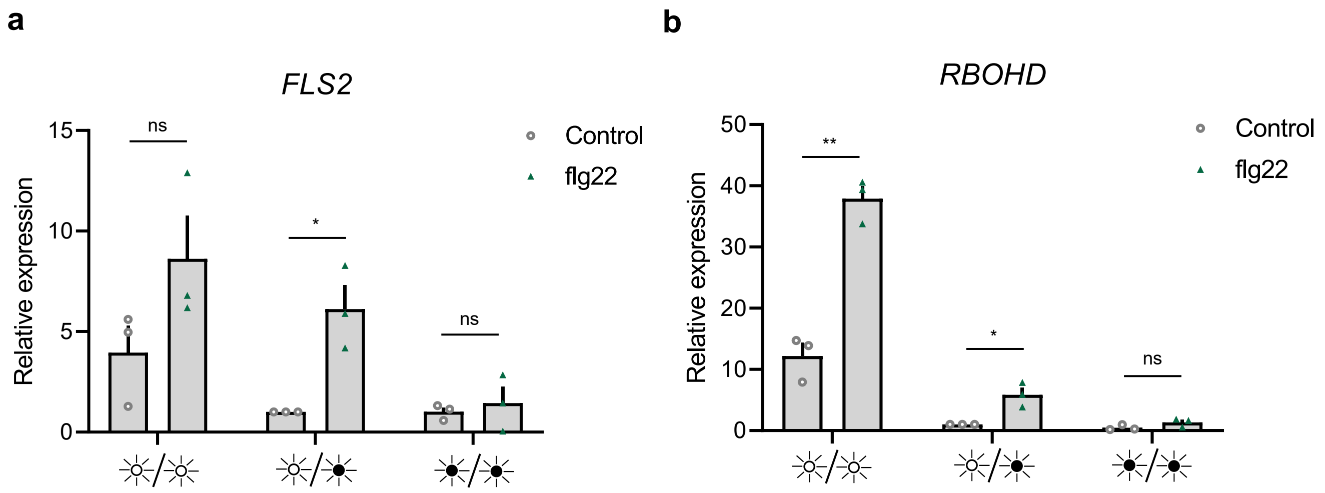

Supplementary Figure 1 | Expression of PTI-associated genes encoding plasma-membrane localized proteins under different light regimes

a-b, Expression levels, as determined by qRT-PCR, of *FLS2* (**a**) and *RBOHD* (**b**) in WT *Arabidopsis* plants that were subjected to 24 hours of the indicated light cycles prior to being infiltrated with flg22 (1 μM). Samples were harvested 6 hours post flg22 treatment. Asterisks indicate statistically significant differences compared to control. ns = non-significant, *p < 0.05, *p < 0.005 Student's T-test. Statistical analysis was performed on values in a and b, obtained from control treated plants in the different light regimes. Statistically significant differences were found between LL versus LD/DD for *RBOHD*, but not *FLS2* (p < 0.05, ANOVA followed by Tukey's range test). Bars and error bars represent mean ± S.E.M. All statistical tests are two-sided.

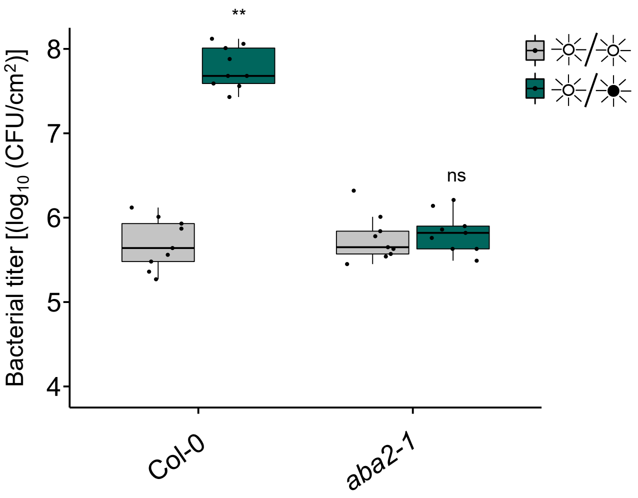

Supplementary Figure 2 | Impact of ABA on darkness-induced virulence
Bacterial titers from WT *Arabidopsis* and *aba2-1* mutant plants syringe-infiltrated with *Pst* DC3000 (1 x 10^5 CFM/ml) at 3 dpi, under the indicated light regimes. Asterisks indicate statistically significant differences compared to control. ns = non-significant, Student's t-test (*aba2-1*) or Wilcoxon-Mann Whitney test (Col-0). In box plots, box edges delineate lower and upper quartiles, the centre line represents the median, and whiskers extend to 1.5 times the interquartile range (IQR). All statistical tests are two-sided.

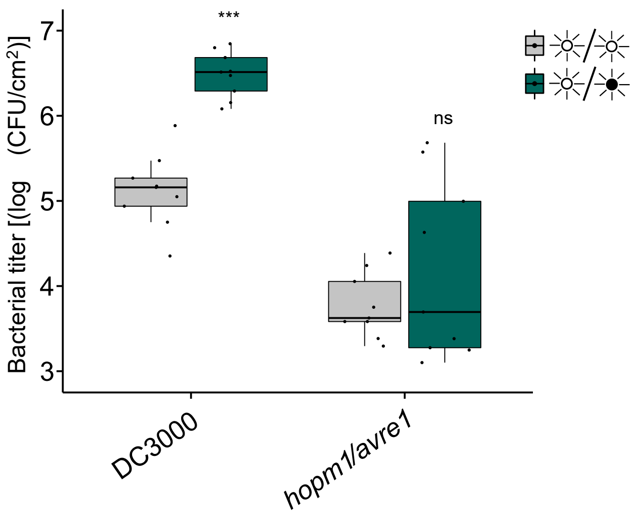

Supplementary Figure 3 | Contributions of HopM1 and AvrE1 to disease progression require darkness
Bacterial titers from WT *Arabidopsis* plants syringe-infiltrated with *Pst* DC3000 (1 x 10^5 CFU/ml) and the water-soaking effector mutant *Pst hopm1⁻/avre1⁻* at 3 dpi under the indicated light regimes. ns = non-significant, *** p < 0.0001, Student's t-test (DC3000) or Wilcoxon-Mann Whitney test (*hopm1⁻/avre1⁻*). In box plots, box edges delineate lower and upper quartiles, the centre line represents the median, and whiskers extend to 1.5 times the interquartile range (IQR). All statistical tests are two-sided.

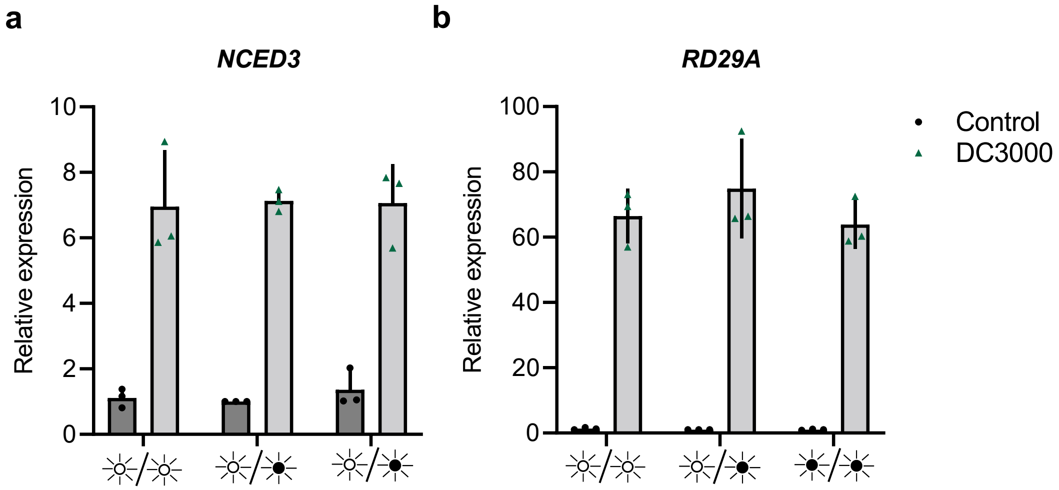

Supplementary Figure 4 | Infection-induced ABA biosynthesis and signaling is not affected by light regimes
a-b, Expression levels, as determined by qRT-PCR, of ABA biosynthesis (*NCED3*) and signaling (*RD29A*) marker genes in WT *Arabidopsis* plants, kept under the indicated light regimes, infected with *Pst* DC3000 (1 x 10^8 CFU/ml) or $MgCl_2$ 10 mM (control) at 24 hpi. Bars and error bars represent mean ± S.E.M. All statistical tests are two-sided.

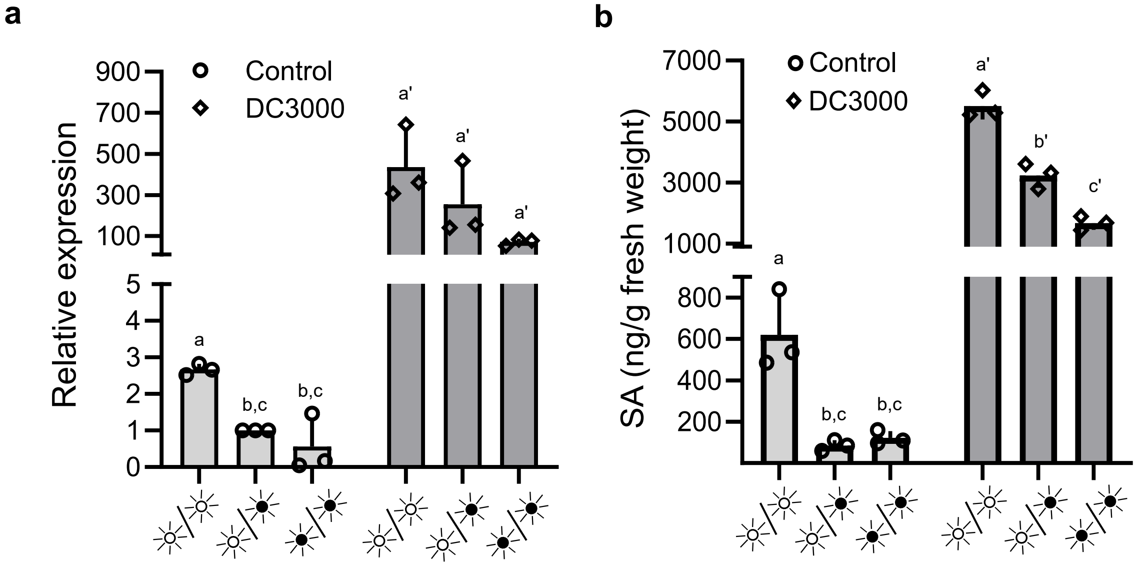

Supplementary Figure 5 | Salicylic acid induction and accumulation in different light regimes under pathogen attack

a, Expression levels, measured by qRT-PCR, of the SA biosynthesis (*ICS1*) marker genes in WT Arabidopsis plants infiltrated with *Pst* DC3000 (1 x 10^8 CFU/ml) after having previously been placed in the identified light settings. Samples were harvested at 24 hpi. **b**, Quantification of SA in WT Arabidopsis plants mock inoculated with $MgCl_2$ 10 mM (control) or infiltrated with *Pst* DC3000 (1 x 10^8 CFU/ml) under the same experimental settings as in a measured by UPLC-MS at 24 hpi. Difference letters indicate statistically significant differences compared to LD ($p < 0.05$, ANOVA followed by Tukey's range test). Bars and error bars represent mean ± S.E.M. All statistical tests are two-sided.

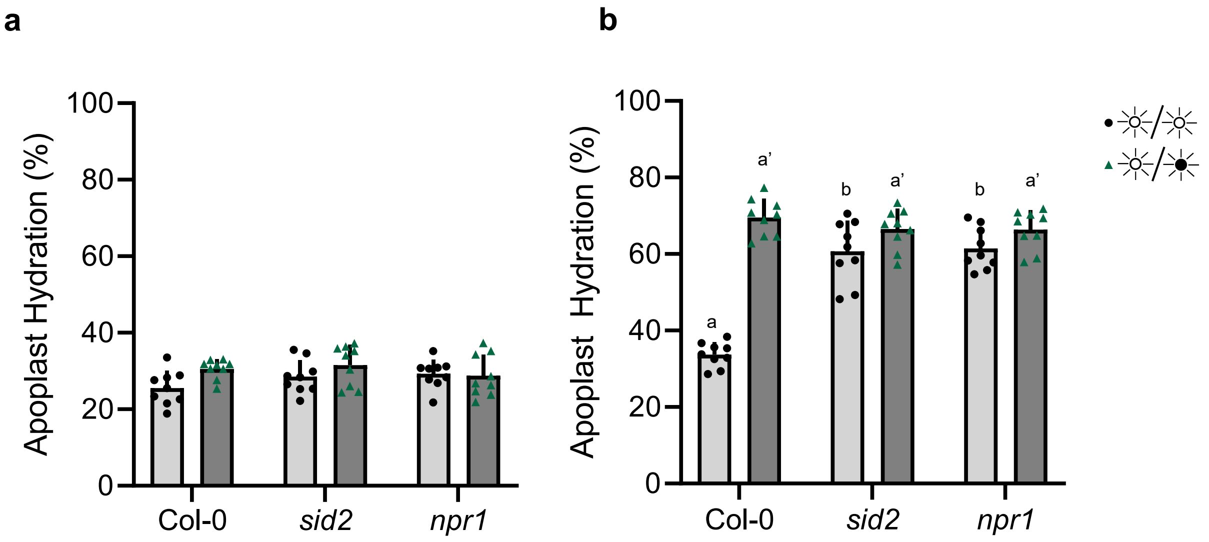

Supplementary Figure 6 | Apoplast hydration in SA biosynthesis and signaling mutant plants under different light regimes

a-b, Apoplast hydration levels at 24 hpi measured from four-week-old *Arabidopsis* WT, *sid2* and *npr1* mutant plants, as indicated, infiltrated with 10 mM $MgCl_2$ (**a**) or with 1 x 10^8 CFU/ml of *Pst* DC3000 (**b**). Plants were kept under constant light for the entire 24-hour period or kept under a 12 hour light/dark regime. Different letters indicate statistically significant differences, $p < 0.05$, ANOVA followed by Tukey's range test. Statistical analysis was performed between the corresponding genotypes/treatments in a and b ($MgCl_2$ versus DC3000) and statistically significant differences were found in all cases ($p < 0.05$, ANOVA followed by Tukey's range test). Bars and error bars represent mean ± S.E.M. All statistical tests are two-sided.

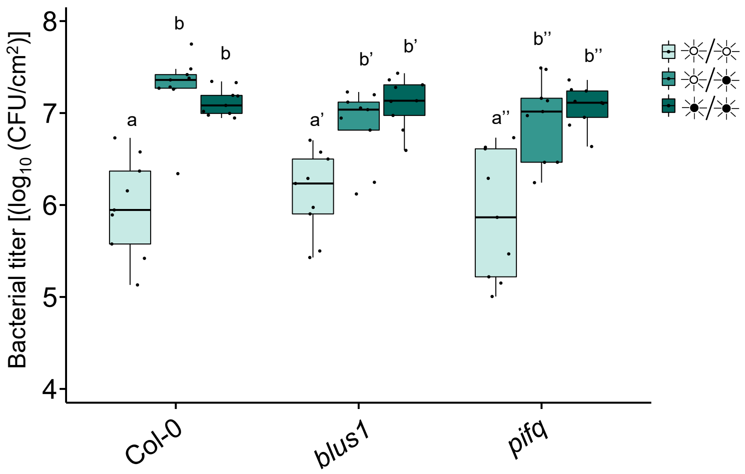

Supplementary Figure 7 | *Pst* virulence in *Arabidopsis* light perception and integration mutants
Bacterial titers in *Arabidopsis* WT, *blus1* and *pifq* mutant plants syringe-infiltrated with *Pst* DC3000 (1 x 10^5 CFU/ml) at 3 dpi under the associated light regimes. Different letters indicate statistically significant differences, $p < 0.05$, Kruskal-Wallis test. In box plots, box edges delineate lower and upper quartiles, the centre line represents the median, and whiskers extend to 1.5 times the interquartile range (IQR). All statistical tests are two-sided.

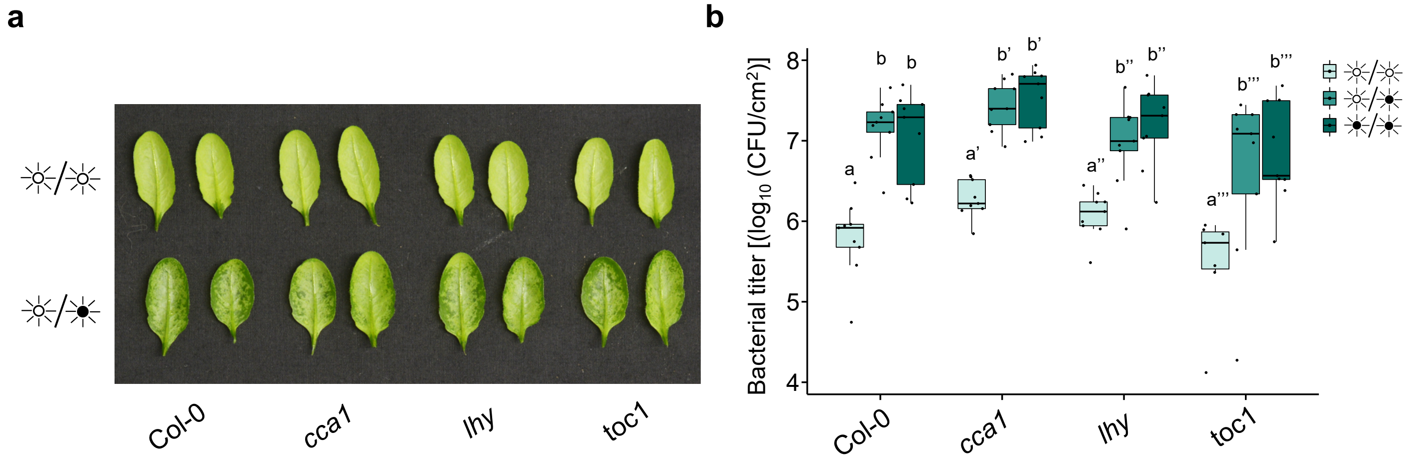

Supplementary Figure 8 | Circadian clock mutants are not affected in water-soaking lesions under constant light

a, Water-soaking phenotypes of *Arabidopsis* WT, *cca1*, *lhy* and *toc1* mutant plants syringe-infiltrated with *Pst* DC3000 (1 x 10^8 CFU/ml) under the indicated light settings. Photos were taken at 24 hpi. **b**, Bacterial titers from *Arabidopsis* WT, *cca1*, *lhy* and *toc1* mutant plants syringe-infiltrated with *Pst* DC3000 (1 x 10^5 CFU/ml) under the indicated light settings at 3 dpi. Different letters indicate statistically significant differences, $p < 0.05$, ANOVA (Col-0, *lhy*) or Krukal-Wallis test (*cca1*, *toc1*). In box plots, box edges delineate lower and upper quartiles, the centre line represents the median, and whiskers extend to 1.5 times the interquartile range (IQR). All statistical tests are two-sided.

Supplementary Table 1. Oligos used in this study

Name	Sequence	Target gene	Application
FRK1-F	5'-GACCGTATATGGACACCGCGTA-3'	FRK1 (AT2G19190)	qRT-PCR
FRK1-R	5'-TTCGCGCTGTTTCTGCAGTG-3'	FRK1 (AT2G19190)	qRT-PCR
WRKY29-F	5'-GCGTAAATACGGGCAGAAAC-3'	WRKY29 (AT4G23550)	qRT-PCR
WRKY29-R	5'-GGTTTGGGTTGGGAAGTTTT-3'	WRKY29 (AT4G23550)	qRT-PCR
ICS1-F	5'-TTTTTTGGTGGCGAGGAGAG-3'	ICS1 (AT1G74710)	qRT-PCR
ICS1-R	5'-CCCCAAGACCCTTTTCGACTA-3'	ICS1 (AT1G74710)	qRT-PCR
PR1-F	5'-CGGAGCTACGCAGAACAACT-3'	PR1 (AT2G14610)	qRT-PCR
PR1-R	5'-CTCGCTAACCCACATGTTCAC-3'	PR1 (AT2G14610)	qRT-PCR
NCED3-F	5'-GGAGAAGGAGGAGAGGAAGA-3'	NCED3 (AT3G14440)	qRT-PCR
NCED3-R	5'-CGACCTGCTTCGCCAAATCAT-3'	NCED3 (AT3G14440)	qRT-PCR
RD29A-F	5'-CGGTTTAGGAGCTCCGTTGG-3'	RD29A (AT5G52310)	qRT-PCR
RD29A-R	5'-GCCTCACCGTATCCAGGTCT-3'	RD29A (AT5G52310)	qRT-PCR
FLS2-F	5'-AGCGCACGACAACATCTTCTTACCG-3'	FLS2 (AT5G46330)	qRT-PCR
FLS2-R	5'-ATCTCGCCAGTCATTTGGTTGTGAG-3'	FLS2 (AT5G46330)	qRT-PCR
RBOHD-F	5'-CTCATTGCCATGCTTCAGTC-3'	RBOHD (AT5G47910)	qRT-PCR
RBOHD-R	5'-TTCCTGGCATTCCACAGTAG-3'	RBOHD (AT5G47910)	qRT-PCR
ACT2-F	5'-CTTGCACCAAGCAGCATGAA-3'	ACT2 (AT3G18780)	qRT-PCR
ACT2-R	5'-CCGATCCAGACACTGTACTTCCTT-3'	ACT2 (AT3G18780)	qRT-PCR
